# Can the use of solid fuels increase the burden of multisite pain among middle-aged and older adults in China?

**DOI:** 10.3389/fpubh.2026.1763838

**Published:** 2026-04-30

**Authors:** ZhiGuo He, Xiong Chen

**Affiliations:** Department of General Surgery, The Fourth Hospital of Changsha (Integrated Traditional Chinese and Western Medicine Hospital of Changsha, Changsha Hospital of Hunan Normal University), Changsha, China

**Keywords:** CHARLS, cooking, heating, multisite pain burden disorders, solid fuel

## Abstract

**Background:**

The relationship between indoor fuel use and multisite body pain remains unclear. This study aims to explore the potential association between solid fuel use and multisite pain burden disorders (MPBD).

**Methods:**

This study was based on data from the China Health and Retirement Longitudinal Study (CHARLS) and conducted a longitudinal analysis of Chinese adults aged 45 years and older, with an average follow-up period of 6.8 years. Indoor fuel use was categorized into solid fuels and clean fuels, and further distinguished between heating and cooking applications. Participants reporting pain in more than seven body sites were defined as having MPBD. Cox regression was used to assess the hazard ratios (HR) and 95% confidence intervals (CIs) associated with fuel use and fuel switch.

**Results:**

During the follow-up period, there were 1,201 (26.17%) cases of MPBD in the heating analysis and 1,525 (25.31%) cases in the cooking analysis. Compared to using clean fuels, the use of solid fuels for heating (HR = 1.21, 95% CI = 1.02–1.44) or cooking (HR = 1.20, 95% CI = 1.04–1.38) was associated with a higher risk of MPBD. Additionally, using solid fuels for both heating and cooking corresponded to an even higher risk (HR = 1.24, 95% CI = 1.00–1.53). In the fuel-switching analysis, compared with consistently using clean fuel, consistently using solid fuel for cooking (HR = 1.41, 95% CI: 1.22–1.62) was associated with a higher risk of MPBD. Compared with consistently using solid fuel, switching to clean fuel (HR = 0.80, 95% CI: 0.67–0.95) and consistently using clean fuel (HR = 0.71, 95% CI: 0.62–0.82) were associated with a lower risk of MPBD. No significant association was observed for heating fuel after full adjustment for covariates.

**Conclusion:**

The use of solid fuels for heating or cooking was associated with a higher risk of MPBD, while switching to clean fuels may help reduce this risk.

## 1 Introduction

Pain can cause persistent discomfort, significantly reducing people’s quality of life and work efficiency, while also consuming a large amount of healthcare resources (1). Pain is generally categorized into acute and chronic pain. Acute pain is usually a symptom of certain diseases or the body’s response to tissue injury and inflammation, playing an important role in the healing process. Pain that persists beyond the acute phase is considered chronic pain (2). Chronic pain is a disease in itself, rather than just a symptom, and requires professional treatment (3, 4). It severely affects the quality of life, with patients often seeking a fundamental solution (5). Globally, over 30% of people are affected by chronic pain, drawing the attention of many researchers (6–8).

Household air pollution (HAP) is primarily caused by the indoor burning of biomass fuels, such as solid fuel use and secondhand smoke (9). HAP contributes to approximately 3 million deaths annually, with low- and middle-income countries being the most affected (10). Additionally, certain areas in high-income countries also use wood for heating (9). Therefore, the global impact of HAP is significant. HAP increases household medical expenses and reduces the level of living consumption (11). In China, air pollution is a serious public health issue. Despite extensive emission-reduction efforts, exposure levels to air pollution in both the environment and households remain high (12). About 81% of the Chinese population still lives in areas where fine particulate matter (PM2.5) concentrations exceed the World Health Organization’s air quality guidelines, and PM2.5 has been responsible for millions of premature deaths worldwide (13). Given this grim situation, combined with China’s large population and the accelerating pace of aging, countless Chinese scholars have conducted extensive research on this topic. The findings indicate that the use of solid fuels increases the risk of respiratory diseases such as asthma, chronic obstructive pulmonary disease, and lung cancer (14, 15); induces hypertension, heart disease, and stunted growth in children (16–18); and can even lead to depression, anxiety, and cognitive impairment (19, 20).

It is evident that HAP can harm human health through biological, psychological, and social factors. Similarly, scholars generally believe that chronic pain is closely related to these factors, such as depression, anxiety, insomnia, traumatic stress, and adverse social conditions (21). However, the relationship between environmental pollution and multisite pain is often overlooked, with few studies exploring the connection between household fuel use and bodily pain. Some studies have found that indoor wood burning can increase the risk of eye pain, headaches, and back pain (22). Other scholars have discovered that the use of solid fuels can increase the incidence of arthritis (23), which is closely linked to joint pain in the shoulders, back, hands, and feet. Nevertheless, most previous research has focused only on the impact of household fuel use on specific diseases or types of pain, without considering its effect on overall body pain burden. Moreover, it remains unknown whether transitioning from solid fuels to clean fuels can reduce the risk of bodily pain. Given the widespread use of solid fuels among the older adults in China and the severe impact of multisite pain, investigating the correlation between these factors is crucial.

This study utilized the CHARLS database, which surveyed individuals aged 45 and above, conducting a longitudinal analysis with 2011 baseline data and four subsequent waves of follow-up. We employed Cox regression to explore the risk of multisite pain burden disorders (MPBD) associated with using solid fuels for heating or cooking compared to using clean fuels, while also assessing whether fuel switching impacts this risk. Lastly, we evaluated the risk of multisite pain in 15 different body parts associated with the use of solid fuels for heating or cooking.

## 2 Methods

### 2.1 Study design and the participants

The CHARLS is a survey targeting Chinese adults aged 45 and older, collecting data on demographics, socioeconomic status, and personal health. The study began with a baseline survey in 2011, followed by systematic follow-up every 2–3 years. The baseline survey used a multi-stage stratified sampling method covering 150 counties/districts and 450 villages/neighborhoods across the country, including 10,257 households with 17,708 participants, providing a nationally representative dataset of older adults in China. Further details are available at http://charls.pku.edu.cn/. CHARLS was approved by the Peking University Biomedical Ethics Committee (approval number: IRB 00001052-11015), and all participants provided informed consent, ensuring there are no ethical issues.

**Figure 1:**
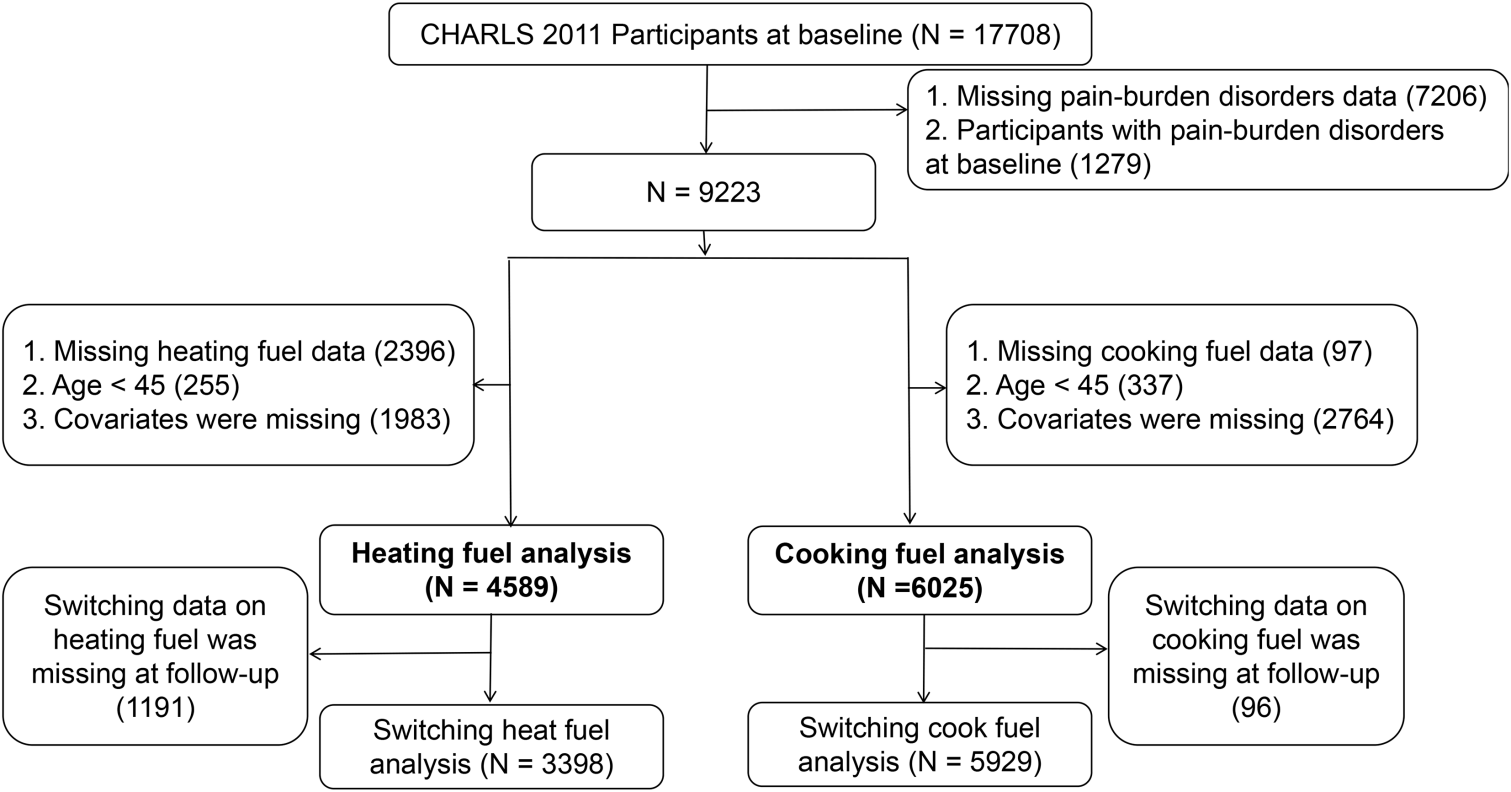
Flowchart for the inclusion process of the study population in heating and cooking analyses.

This study retrospectively analyzed five waves of CHARLS data from 2011 to 2020 (wave1-2011, wave2-2013, wave3-2015, wave4-2018, wave5-2020), with wave 1 serving as the baseline data and the remaining four as follow-up data. A total of 17,708 participants were included at baseline, with the following inclusion criteria: (1) participants with missing MPBD data at baseline or during follow-up were excluded (*n* = 7,206); (2) participants diagnosed with MPBD at baseline were excluded (*n* = 1,279); (3) participants under 45 years of age, those with missing covariates, and those with missing data on heating or cooking fuel at baseline were excluded (heating *n* = 4,634; cooking *n* = 3,198). Ultimately, 4,589 and 6,025 participants were included in the heating and cooking fuel studies, respectively. Furthermore, to explore the impact of solid or clean fuels on MPBD, participants with missing fuel switch data during follow-up were excluded, leaving 3,398 and 5,929 participants for the fuel switch analysis. After excluding participants with missing values for the included variables, the average follow-up period was 6.8 years. The detailed selection process is shown in Figure 1.

### 2.2 Assessment of household fuel types

The household fuel data was obtained from the housing characteristic section of the CHARLS survey. Participants were asked during the interview, “What is the main energy source for heating?” and “What is the main energy source for cooking?” According to this standardized questionnaire, heating energy sources were categorized into clean fuels (solar energy, piped natural gas, liquefied petroleum gas, or electricity) and solid fuels (coal, straw, firewood). Similarly, cooking energy sources were divided into clean fuels (piped natural gas, biogas, liquefied petroleum gas, or electricity) and solid fuels (coal, straw, firewood). We combined the heating and cooking fuel data and classified four mixed fuel usage types: clean fuel for both heating and cooking, solid fuel for heating and clean fuel for cooking, clean fuel for heating and solid fuel for cooking, and solid fuel for both heating and cooking. Additionally, fuel switching was determined by comparing the fuel used at the baseline with that at the follow-up. If the fuel used at the endpoint differed from the baseline, it was defined as fuel switching. Similarly, fuel switching was divided into four types: always using clean fuel, switching from clean fuel to solid fuel, switching from solid fuel to clean fuel, and always using solid fuel.

### 2.3 Assessment of MPBD and body pain

In this study, baseline (2011) was defined as the starting point of follow-up, and event time was defined as the interview date of the wave in which the first event occurred. We used bodily pain burden as the primary outcome and applied right censoring; participants who did not experience a pain burden event by the last follow-up (2020) were censored. Participants were asked during the interview whether they experienced pain in 15 different body parts (head, shoulders, arms, wrists, fingers, chest, stomach, back, waist, buttocks, legs, knees, ankles, toes, neck). Based on their responses, we assigned 1 point for each type of pain burden, with a total score of 15 points. If the total pain burden score was ≥7, it was defined as MPBD. To assess whether the analytical results were influenced by the choice of thresholds, we conducted sensitivity analyses by comparing different cutoff values (e.g., ≥4, ≥7, and ≥10). Additionally, we treated each of the 15 types of bodily pain as separate outcomes and applied new inclusion and exclusion criteria to the study population (Supplementary Figure S1) to explore the impact of heating or cooking fuel on pain in different body parts.

### 2.4 Covariates

Based on previous research, we included the needed covariates (17, 24). Demographic variables include age, gender (male and female), hukou (urban and rural), per capita household consumption (<7,700 and ≥7,700 yuan), education (<high school and ≥high school), and marital status (unmarried and married). Other variables include BMI (underweight: <18.5, normal: 18.5–24, overweight: 24–28, and obesity: ≥28), smoking (smoke and no smoke), alcohol consumption (drink and no drink), hypertension (yes and no), diabetes (yes and no), heart disease (yes and no), and lung disease (yes and no). Hypertension was diagnosed based on two survey questions: “Doctor-diagnosed health problems: Ever had high blood pressure?” or “Do you take any medication for high blood pressure?” If either question was answered “yes,” the participant was classified as having hypertension.

### 2.5 Statistical

The baseline characteristics of the study population were described using means (percentages), while the Wilcoxon rank-sum test and Pearson’s chi-squared test were used to differentiate between continuous and categorical variables. Hazard ratios (HRs) and 95% confidence intervals (CIs) were estimated using Cox proportional hazards regression models, with cluster-robust standard errors applied at the level of the primary sampling units (communities). To assess the risk of pain burden associated with different types of fuel use, we used follow-up time (in years) as the time scale, with MPBD as the endpoint event, and applied Cox regression to explore the relationship between heating or cooking fuel types, fuel switch, and MPBD. In addition, we conducted an exploratory analysis of the associations between solid fuel use and the risk of pain across 15 different body sites. Age, gender, hukou, household consumption, education, marital status, BMI, smoking, drinking, hypertension, diabetes, heart disease, and lung disease were included as covariates in the model adjustment. To verify the robustness of our study findings, we conducted a series of sensitivity analyses. Multiple imputation and inverse probability of censoring weighting (IPCW) were used to assess whether missing data could introduce bias into the primary analysis. The midpoint imputation between waves, proportional hazards assumption testing, and time-interaction analyses were applied to evaluate the impact of event-time assignment and censoring. Survey-weighted models and cluster-robust standard errors were employed to validate the accuracy of the main analysis. Additionally, E-values were calculated to assess the potential influence of unmeasured confounding on our results.

All analyses were conducted using R software version 4.5.0. A two-tailed test was used to determine statistical significance, with significance defined as P less than 0.05.

## 3 Results

### 3.1 Basic characteristics of the study population

As shown in Table 1, a total of 4,589 and 6,025 participants were included in the analyses of heating fuel and cooking fuel, respectively, at baseline. Participants who used solid fuels for heating or cooking were generally older, had lower household consumption levels, lower education levels, and most had rural household registration and a normal BMI. Differences included a higher proportion of smokers, fewer participants with diabetes, and more participants with lung disease among those using solid fuels for cooking. Notably, participants using solid fuels for heating and cooking had a higher pain burden at the end of the follow-up period. In addition, we compared the distributions of key baseline variables between the excluded and included groups. Apart from differences in some demographic characteristics, there were no significant differences in important indicators such as age, hypertension, diabetes, heart disease, and pain burden (Supplementary Table S9).

**Table 1:** Baseline characteristics of participants categorized by fuel type usage.

Variable	Heat				Cook			
	Overall, *N* = 4,589^1^	Clean fuel, *N* = 1,054^1^	Solid fuel, *N* = 3,535^1^	*p* value^2^	Overall, *N* = 6,025^1^	Clean fuel, *N* = 2,471^1^	Solid fuel, *N* = 3,554^1^	*p* value^2^
Age (years)	57 (51, 63)	56 (49, 62)	57 (51, 63)	<0.001	57 (51, 63)	56 (49, 62)	58 (52, 64)	<0.001
Gender				0.6				0.7
Male	2,104 (46%)	491 (47%)	1,613 (46%)		2,765 (46%)	1,141 (46%)	1,624 (46%)	
Female	2,485 (54%)	563 (53%)	1,922 (54%)		3,260 (54%)	1,330 (54%)	1,930 (54%)	
Hukou				<0.001				<0.001
Urban	615 (13%)	282 (27%)	333 (9.4%)		953 (16%)	702 (28%)	251 (7.1%)	
Rural	3,974 (87%)	772 (73%)	3,202 (91%)		5,072 (84%)	1,769 (72%)	3,303 (93%)	
Household consumption				<0.001				<0.001
<7,700	3,447 (75%)	637 (60%)	2,810 (79%)		4,423 (73%)	1,575 (64%)	2,848 (80%)	
>=7,700	1,142 (25%)	417 (40%)	725 (21%)		1,602 (27%)	896 (36%)	706 (20%)	
Education				<0.001				<0.001
<High school	4,150 (90%)	911 (86%)	3,239 (92%)		5,377 (89%)	2,056 (83%)	3,321 (93%)	
>=High school	439 (9.6%)	143 (14%)	296 (8.4%)		648 (11%)	415 (17%)	233 (6.6%)	
Marry				0.7				0.6
Unmarried	412 (9.0%)	92 (8.7%)	320 (9.1%)		558 (9.3%)	235 (9.5%)	323 (9.1%)	
Married	4,177 (91%)	962 (91%)	3,215 (91%)		5,467 (91%)	2,236 (90%)	3,231 (91%)	
BMI				<0.001				<0.001
Underweight	269 (5.9%)	47 (4.5%)	222 (6.3%)		344 (5.7%)	115 (4.7%)	229 (6.4%)	
Normal	2,412 (53%)	518 (49%)	1,894 (54%)		3,151 (52%)	1,185 (48%)	1,966 (55%)	
Overweight	1,374 (30%)	363 (34%)	1,011 (29%)		1,815 (30%)	829 (34%)	986 (28%)	
Obesity	534 (12%)	126 (12%)	408 (12%)		715 (12%)	342 (14%)	373 (10%)	
Smoking status				0.080				<0.001
Non-smoker	3,187 (69%)	755 (72%)	2,432 (69%)		4,226 (70%)	1,791 (72%)	2,435 (69%)	
Smoke	1,402 (31%)	299 (28%)	1,103 (31%)		1,799 (30%)	680 (28%)	1,119 (31%)	
Drinking status				0.2				0.3
Non-drinker	3,759 (82%)	849 (81%)	2,910 (82%)		4,965 (82%)	2,050 (83%)	2,915 (82%)	
Drink	830 (18%)	205 (19%)	625 (18%)		1,060 (18%)	421 (17%)	639 (18%)	
Hypertension				0.3				0.2
No	3,455 (75%)	782 (74%)	2,673 (76%)		4,514 (75%)	1,832 (74%)	2,682 (75%)	
Yes	1,134 (25%)	272 (26%)	862 (24%)		1,511 (25%)	639 (26%)	872 (25%)	
Diabetes				0.3				<0.001
No	4,362 (95%)	995 (94%)	3,367 (95%)		5,714 (95%)	2,313 (94%)	3,401 (96%)	
Yes	227 (4.9%)	59 (5.6%)	168 (4.8%)		311 (5.2%)	158 (6.4%)	153 (4.3%)	
Heart disease				0.9				0.2
No	4,125 (90%)	949 (90%)	3,176 (90%)		5,430 (90%)	2,211 (89%)	3,219 (91%)	
Yes	464 (10%)	105 (10.0%)	359 (10%)		595 (9.9%)	260 (11%)	335 (9.4%)	
Lung disease				0.4				<0.001
No	4,207 (92%)	973 (92%)	3,234 (91%)		5,546 (92%)	2,311 (94%)	3,235 (91%)	
Yes	382 (8.3%)	81 (7.7%)	301 (8.5%)		479 (8.0%)	160 (6.5%)	319 (9.0%)	
pain_burden				<0.001				<0.001
No	3,388 (74%)	826 (78%)	2,562 (72%)		4,500 (75%)	1,930 (78%)	2,570 (72%)	
Yes	1,201 (26%)	228 (22%)	973 (28%)		1,525 (25%)	541 (22%)	984 (28%)	

1 Median (IQR); *n* (%).

2 Wilcoxon rank sum test; Pearson’s Chi-squared test.

### 3.2 Association between baseline household fuel use and MPBD risk

As shown in Supplementary Table S1, during the follow-up period, 1,201 (26.17%) and 1,525 (25.31%) cases of MPBD occurred in the heating and cooking analysis, respectively. Most of these individuals were female, had rural residency, lower consumption levels, lower education levels, and lower rates of smoking and drinking, while higher rates of hypertension, heart disease, and lung disease were observed. Moreover, data from all four follow-up waves consistently showed a significant association between different types of fuel use and multisite bodily pain, with solid fuel users exhibiting a higher prevalence of pain (Supplementary Tables S2–S5).

Univariate Cox regression showed that being female, living in rural areas, having hypertension, heart disease, and lung disease were all risk factors for MPBD, while higher education levels and smoking or drinking habits were possibly associated with a lower risk of MPBD. Additionally, unlike the heating analysis, higher household consumption levels in the cooking analysis seemed to correspond to a lower risk of MPBD (Supplementary Table S6).

Using longitudinal data from the 2011–2020 follow-up, we first explored the relationship between baseline household heating and cooking fuel use and the risk of MPBD. The results showed that even after adjusting for covariates, using solid fuels for heating was associated with a higher risk of MPBD compared to clean fuels (HR = 1.21, 95% CI = 1.02–1.44, *p* = 0.032). Similarly, using solid fuels for cooking was also associated with a higher risk (HR = 1.20, 95% CI = 1.04–1.38, *p* = 0.010) (Table 2).

**Table 2:** Hazard ratios of MPBD associated with using solid fuel for heating or cooking.

Characteristic	Model 1			Model 2			Model 3		
	HR^1^	95% CI^1^	*p*-value	HR^1^	95% CI^1^	*p*-value	HR^1^	95% CI^1^	*p*-value
heat_fuel									
Clean fuel heat	–	–		–	–		–	–	
Solid fuel heat	1.34	1.13, 1.59	**<0.001**	1.24	1.04, 1.48	**0.019**	1.21	1.02, 1.44	**0.032**
cook_fuel									
Clean fuel cook	–	–		–	–		–	–	
Solid fuel cook	1.33	1.16, 1.53	**<0.001**	1.21	1.05, 1.40	**0.008**	1.20	1.04, 1.38	**0.010**

1 HR = hazard ratio, CI = confidence interval.

Model 1: Unadjusted. Model 2: Adjusted for age, gender, hukou, household consumption, education, and marital status. Model 3: Adjusted for age, gender, hukou, household consumption, education, marital status, BMI, smoking, drinking, hypertension, heart disease, diabetes, and lung diseases. *p* < 0.05 is highlighted in bold. Cluster-robust standard errors were applied at the level of the primary sampling units (communities).

Next, to further investigate the impact of fuel choices on MPBD, we combined the two categories of heating and cooking fuels. Cox regression, using clean fuel for both heating and cooking as the reference, revealed that in the fully adjusted model, using solid fuel for both heating and cooking was associated with a significantly higher risk of MPBD (HR = 1.24, 95% CI = 1.00–1.53, *p* = 0.045) (Table 3). This suggests that household solid fuel use may contribute to the onset of physical pain.

**Table 3:** Hazard ratios of MPBD associated with mixed heating and cooking fuel use.

Characteristic	Model 1			Model 2			Model 3		
	HR^1^	95% CI^1^	*p*-value	HR^1^	95% CI^1^	*p*-value	HR^1^	95% CI^1^	*p*-value
heat_and_cook									
Clean fuel heat and cook	–	–		–	–		–	–	
Solid fuel heat and clean fuel cook	1.11	0.89, 1.38	0.4	1.05	0.83, 1.32	0.7	1.03	0.82, 1.29	0.8
Clean fuel heat and solid fuel cook	1.03	0.75, 1.42	0.8	0.91	0.66, 1.26	0.6	0.91	0.66, 1.27	0.6
Solid fuel heat and cook	1.43	1.17, 1.75	**<0.001**	1.27	1.02, 1.57	**0.031**	1.24	1.00, 1.53	**0.045**

1 HR = hazard ratio, CI = confidence interval.

Model 1: Unadjusted. Model 2: Adjusted for age, gender, hukou, household consumption, education, and marital status. Model 3: Adjusted for age, gender, hukou, household consumption, education, marital status, BMI, smoking, drinking, hypertension, heart disease, diabetes, and lung diseases. *p* < 0.05 is highlighted in bold. Cluster-robust standard errors were applied at the level of the primary sampling units (communities).

### 3.3 Association between fuel switching and MPBD risk

To investigate whether fuel switching is associated with the risk of MPBD, we defined exposure as the status at the beginning of each time interval and used the wave in which the event occurred as the event time, treating fuel use as a time-varying variable in the Cox proportional hazards model. Heating and cooking fuel use were categorized, with “consistently using clean fuel” and “consistently using solid fuel” serving as reference groups in the time-varying Cox regression analyses.

In the fully adjusted model, compared with consistently using clean fuel, consistently using solid fuel for cooking (HR = 1.41, 95% CI: 1.22–1.62, *p* < 0.001) was associated with a higher risk of MPBD. Compared with consistently using solid fuel, switching to clean fuel (HR = 0.80, 95% CI: 0.67–0.95, *p* = 0.013) and consistently using clean fuel (HR = 0.71, 95% CI: 0.62–0.82, *p* < 0.001) were associated with a lower risk of MPBD. However, for heating fuel, no statistically significant associations were observed after full adjustment for covariates (Table 4).

**Table 4:** Hazard ratios for MPBD associated with fuel switching categorized by heating and cooking.

Characteristic	Model 1			Model 2			Model 3		
	HR^1^	95% CI^1^	*p*-value	HR^1^	95% CI^1^	*p*-value	HR^1^	95% CI^1^	*p*-value
heat_status									
always_clean	–	–		–	–		–	–	
switched_to_clean	1.50	1.14, 1.97	**0.004**	1.25	0.92, 1.70	0.2	1.17	0.84, 1.65	0.4
switched_to_solid	1.39	1.05, 1.84	**0.021**	1.15	0.84, 1.56	0.4	1.11	0.78, 1.58	0.6
always_solid	1.52	1.28, 1.81	**<0.001**	1.27	1.05, 1.54	**0.012**	1.22	0.99, 1.51	0.057
heat_status									
always_solid	–	–		–	–		–	–	
switched_to_clean	0.99	0.76, 1.27	>0.9	0.98	0.74, 1.30	0.9	0.96	0.70, 1.31	0.8
switched_to_solid	0.91	0.70, 1.20	0.5	0.90	0.67, 1.21	0.5	0.91	0.65, 1.27	0.6
always_clean	0.66	0.55, 0.78	**<0.001**	0.78	0.65, 0.95	**0.012**	0.82	0.66, 1.01	0.057
cook_status									
always_clean	–	–		–	–		–	–	
switched_to_clean	1.27	1.09, 1.49	**0.002**	1.19	1.01, 1.41	**0.039**	1.13	0.94, 1.35	0.2
switched_to_solid	1.38	1.17, 1.62	**<0.001**	1.26	1.04, 1.51	**0.015**	1.18	0.97, 1.44	0.10
always_solid	1.61	1.42, 1.82	**<0.001**	1.43	1.25, 1.65	**<0.001**	1.41	1.22, 1.62	**<0.001**
cook_status									
always_solid	–	–		–	–		–	–	
switched_to_clean	0.79	0.68, 0.92	**0.002**	0.83	0.71, 0.97	**0.021**	0.80	0.67, 0.95	**0.013**
switched_to_solid	0.86	0.74, 1.00	0.055	0.88	0.74, 1.03	0.11	0.84	0.70, 1.01	0.063
always_clean	0.62	0.55, 0.71	**<0.001**	0.70	0.61, 0.80	**<0.001**	0.71	0.62, 0.82	**<0.001**

1 HR = hazard ratio, CI = confidence interval.

Model 1: Unadjusted. Model 2: Adjusted for age, gender, hukou, household consumption, education, and marital status. Model 3: Adjusted for age, gender, hukou, household consumption, education, marital status, BMI, smoking, drinking, hypertension, heart disease, diabetes, and lung diseases. *p* < 0.05 is highlighted in bold. Cluster-robust standard errors were applied at the level of the primary sampling units (communities).

### 3.4 Association between solid household fuel use and pain in 15 different parts of the body

**Figure 2:**
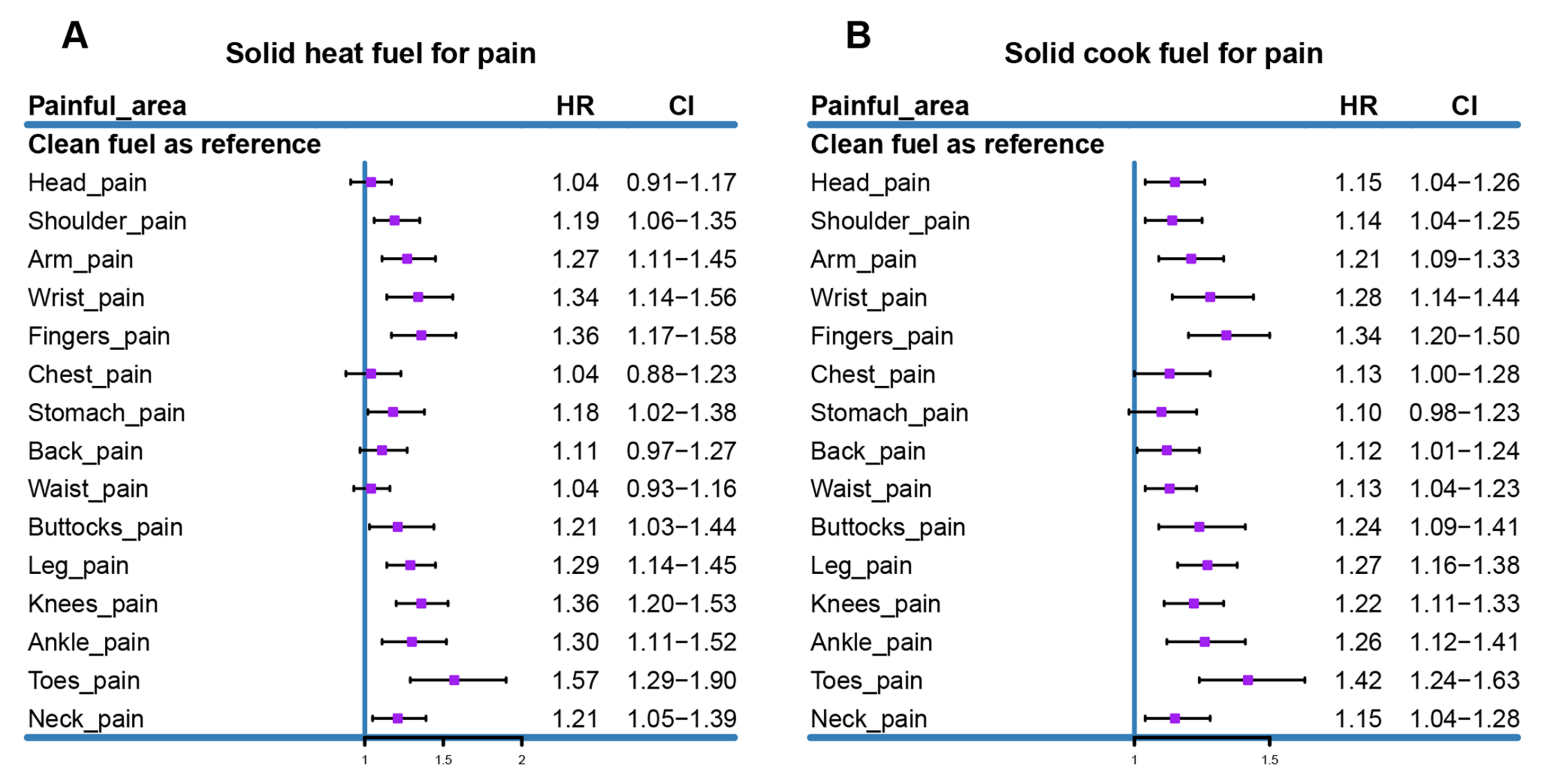
COX regression analysis of solid fuel use on 15 types of pain: **(A)** Hazard ratios for the 15 types of pain associated with using solid fuel for heating. **(B)** Hazard ratios for the 15 types of pain associated with using solid fuel for cooking. Adjusted for age, gender, hukou, household consumption, education, marital status, BMI, smoking, drinking, hypertension, heart disease, diabetes, and lung diseases.

Due to the varying locations of the 15 different types of bodily pain, we aimed to explore the differences in the risk of pain associated with heating or cooking fuel use across these specific areas. Therefore, we included and excluded participants based on heating or cooking fuel use and each of the 15 types of bodily pain (Supplementary Figure S1) and conducted separate Cox regressions with the use of clean fuel as the reference. The results showed that using solid fuel for heating significantly increased the risk of pain in 11 areas: shoulders, arms, wrists, fingers, stomach, buttocks, legs, knees, ankles, toes, and neck. Meanwhile, using solid fuel for cooking significantly increased the risk of pain in 13 areas: head, shoulders, arms, wrists, fingers, back, waist, buttocks, legs, knees, ankles, toes, and neck (Figure 2).

### 3.5 Sensitivity analyses

To verify the robustness and reliability of the study findings, we conducted multiple sensitivity analyses. The results of the sensitivity analyses regarding pain threshold selection indicated that the association between solid fuel use and an increased risk of MPBD remained robust and did not change across different threshold definitions (Supplementary Tables S7, S8). The results from multiple imputation and inverse probability of censoring weighting (IPCW) indicated that the association between solid fuel use for heating and an increased risk of MPBD was highly robust. The hazard ratio for solid fuel use for cooking under multiple imputation (HR = 1.32) may be closer to the true population-level effect. Nevertheless, the core finding that solid fuel use is associated with an elevated risk of MPBD remained robust (Supplementary Table S10).

The midpoint imputation analysis yielded results that were entirely consistent with those from the original time-to-event analysis, indicating that our core findings are not sensitive to the specific method used for event-time assignment (Supplementary Table S11). Testing of the proportional hazards assumption showed a global *p*-value of 0.0756 for heating fuel and 0.0166 for cooking fuel, suggesting a detectable overall violation of the proportional hazards assumption (Supplementary Table S12). To further evaluate the impact of this issue on our conclusions, we incorporated interaction terms between solid fuel use and the logarithm of follow-up time into the Cox model. The results indicated that these interaction terms were not statistically significant (heating fuel: *p* = 0.972; cooking fuel: *p* = 0.971) (Supplementary Table S13). This provides direct evidence that the hazard ratios for solid fuel use do not vary significantly over time, thereby supporting the validity of the proportional hazards assumption. Therefore, although the proportional hazards assumption test suggested a potential minor violation, the time-interaction analysis indicated that this did not alter the core conclusions.

Sensitivity analyses using the standard Cox model, survey-weighted models, and cluster-robust standard errors showed that although the confidence interval for the survey-weighted model of cooking fuel included the null value (HR = 1.17, 95% CI: 0.99–1.38), the HR were highly consistent with those obtained from the other methods, and the association remained statistically significant when using cluster-robust standard errors. Overall, the HR derived from the three approaches were nearly identical for both heating and cooking fuels, and the associations were consistently significant, indicating that the magnitude of the estimated associations is not sensitive to the choice of variance estimation or weighting method (Supplementary Table S14). In addition, to assess the potential impact of unmeasured confounding, we calculated E-values. As shown in Supplementary Table S15, for solid fuel use in heating, the adjusted hazard ratio (HR) was 1.21, with an E-value of 1.14. For solid fuel use in cooking, the adjusted HR was 1.20, with an E-value of 1.14. Although an E-value of 1.14 is not large, it suggests that the observed associations are reasonably robust to weak-to-moderate residual confounding.

## 4 Discussion

Our study is the first to explore the relationship between indoor fuel use and MPBD. Compared with the use of clean fuels, the use of solid fuels for heating or cooking was associated with a higher risk of MPBD. In the fuel-switching analysis, persistent use of solid fuel for cooking was associated with a higher risk of MPBD, whereas switching from solid to clean fuel was associated with a lower risk of MPBD. Finally, in separate Cox regression analyses for 15 types of bodily pain, we found that using solid fuels for heating or cooking was associated with an increased risk of most types of pain. This suggests that indoor solid fuel use may increase the overall pain burden in older adults individuals or cause pain in specific body parts.

HAP has long been a major health hazard impacting disease risk and life expectancy, particularly due to the use of indoor solid fuels. Numerous studies have explored the health effects of HAP, such as the increased risk of depression, heart disease, and sarcopenia associated with solid fuel use (17, 21, 25). However, research on the relationship between solid fuels and physical pain remains limited. Díaz et al. found a link between solid fuel use and eye pain, headaches, and back pain (22), while Deng et al. discovered an association between solid fuel use and arthritis (23). Li et al. reported that household solid fuel use was associated with back pain and neck pain in middle-aged and older adults (26). HAP may even indirectly lead to physical disability by triggering bodily pain (27). In addition, a study of older adults in India found that individuals from households using solid fuels for cooking had a higher likelihood of developing angina (28). Similarly, we found that using solid fuels for heating and cooking significantly increases the risk of MPBD. Moreover, individuals who use solid fuels for both heating and cooking face an even higher risk of MPBD. This aligns with our expectations, indicating that more frequent use or greater exposure to solid fuels may lead to a higher MPBD risk, whereas using clean indoor fuels may effectively reduce this risk.

Univariate COX regression analysis of clinical characteristics showed that being female, living in rural areas, and having hypertension, heart disease, and lung diseases are risk factors for MPBD. This aligns with our observed results, as many rural residents in China primarily use solid fuels for heating and cooking, increasing their exposure opportunities (29). Research indicates that women and children are more susceptible to the effects of HAP, as they are often exposed to the highest levels of pollutants and typically lack the decision-making power regarding solid fuel use (30, 31). Evidence suggests that the use of solid fuels increases the incidence risk of chronic diseases like hypertension (16). A higher burden of chronic illness may make individuals more prone to chronic pain or more sensitive to it. On the other hand, individuals with higher education levels, as well as those who smoke and drink, seem to have a lower risk of MPBD. Those with higher education may have a more comprehensive understanding of the dangers posed by solid fuels and are thus more likely to opt for cleaner fuel alternatives. Regarding smoking and drinking, there is a bidirectional relationship between multisite pain and these behaviors, as chronic pain can stimulate tobacco and alcohol consumption (32, 33). Some researchers suggest that nicotine has direct analgesic effects (34), and alcohol is associated with pain relief (32), which may help explain our findings. However, there is currently no clear evidence that smoking or alcohol consumption alleviates multisite pain, and we do not recommend these behaviors as strategies for pain management. Finally, in the cooking analysis, a higher consumption level was associated with lower risk, possibly because households with better economic capacity can choose more clean fuel options.

In the fuel-switching analysis, using consistently clean fuel as the reference, long-term use of solid fuel for cooking was associated with a higher risk of MPBD. Additionally, using consistently solid fuel as the reference, switching from solid to clean fuel and consistently using clean fuel for cooking were both associated with a lower risk of MPBD, with consistently using clean fuel showing the lowest HR. These findings suggest that, compared with persistent use of solid fuel, switching to clean fuel may reduce the risk, although the risk may still remain higher than that observed with consistently using clean fuel. However, no significant association was observed between heating fuel switching and the risk of MPBD. We speculate that this discrepancy is due to the different patterns of exposure to solid fuels for cooking and heating (35). Cooking occurs daily, while heating is seasonal and regional; for instance, some areas in southern China do not have heating practices, or heating is only used during the cold winter months (36). This could lead to data collection biases, highlighting the need for more comprehensive and in-depth research to validate our findings.

To explore the relationship between pain in 15 different body areas and fuel usage, we conducted separate COX regression analyses. The results indicated that using solid fuels for heating may increase the risk of pain in the shoulders, arms, wrists, fingers, stomach, buttocks, legs, knees, ankles, toes, and neck—11 areas in total. In contrast, using solid fuels for cooking was associated with increased pain in the head, shoulders, arms, wrists, fingers, back, waist, buttocks, legs, knees, ankles, toes, and neck—13 areas in total. This aligns with previous research linking solid fuel use to increased risks of conditions such as hypertension, arthritis, and sarcopenia (16, 23, 25), which in turn can lead to a heavier burden of pain in these specific areas. Furthermore, the burden of pain associated with cooking appears to be greater than that linked to heating, likely due to the differing patterns of exposure to solid fuels, making individuals more susceptible to HAP when cooking.

Research on the specific mechanisms linking solid fuels to human body pain is very limited, leaving us uncertain about the details. The combustion of solid fuels produces various pollutants, primarily including polycyclic aromatic hydrocarbons (PAHs), particulate matter (PM), volatile organic compounds (VOCs), nitrogen dioxide, carbon monoxide, and sulfur dioxide, often at concentrations two to three times higher in indoor environments (37–39). The cytotoxicity of PM2.5 generated from solid fuels is mainly driven by oxidative stress (40). Gas pollutants on respiratory epithelial cells and membranes can trigger oxidative stress (OS), leading to the formation of reactive oxygen species (ROS) and pro-inflammatory mediators (41). The production of ROS can initiate inflammatory responses, affecting multiple body systems and resulting in a state of cellular stress (42). Prolonged exposure to indoor pollution, along with repeated oxidative stress and inflammatory responses, may contribute to multisite body pain. Notably, some studies have found that adequate ventilation can mitigate these harmful effects (43). Therefore, encouraging the transition to clean fuels and improving ventilation systems could be effective in alleviating multisite body pain.

We explored the relationship between indoor fuel use and body pain burden for the first time using the CHARLS database. This study has several advantages: (1) CHARLS is a large, continuously updated nationally representative database that provides reliable analytical results; (2) we considered mixed patterns of heating and cooking fuel use and introduced the concept of fuel transition; (3) both specific pain burdens in different body areas and overall body pain burden were included. However, our study also has limitations: (1) Information on indoor fuel use and body pain burden was obtained from questionnaires, which may lead to recall bias; (2) although we made every effort to adjust for covariates, the potential influence of unknown factors (such as depressive symptoms, physical activity or functional limitations, arthritis or musculoskeletal conditions, occupation or labor intensity, sleep status, and the use of analgesic medications) cannot be ruled out; (3) due to limitations of the CHARLS database, information on kitchen ventilation facilities (such as exhaust fans, range hoods, and window conditions) and cooking stove characteristics was not available. Given the importance of ventilation systems in studies of indoor pollution, incorporating this dimension in future research would be highly valuable; (4) while the sample size was sufficiently large, the exclusion of participants due to missing data may introduce bias; (5) the study population was limited to older adults in China, necessitating research including more diverse racial groups to validate our findings; (6) fuel stacking may occur in some households, and individual exposure levels can be influenced by factors such as ventilation conditions, stove type, and duration of use. These unobserved sources of heterogeneity may lead to exposure misclassification.

We conducted a longitudinal analysis of the middle-aged and older population in China and found that the use of solid fuels for heating or cooking was associated with a higher risk of MPBD, whereas switching to clean fuels was associated with a lower risk. This suggests that encouraging households to use clean fuels for heating and cooking may help alleviate multisite pain burdens among individuals.

## Data Availability

Publicly available datasets were analyzed in this study. This data can be found here: All data used in this study can be accessed at http://charls.pku.edu.cn/.
