# Supplementary material for: Can the use of solid fuels increase the burden of multisite pain among middle-aged and older adults in China?
Source: Front Public Health. 2026 Apr 30;14:1763838. doi: 10.3389/fpubh.2026.1763838 (PMC13171800; doi:10.3389/fpubh.2026.1763838)
Supplement: Supplementary file 1 [file Data_Sheet_1.docx]

**Supplementary Table S1** Baseline (wave1-2011) characteristics of participants in the heating and cooking analysis classified by the occurrence of MPBD during the follow-up period.

|  | **MPBD (heat)** | | | | **MPBD (cook)** | | | |
| --- | --- | --- | --- | --- | --- | --- | --- | --- |
| **Variable** | **Overall**, N = 4,589^1^ | **No**, N = 3,388^1^ | **Yes**, N = 1,201^1^ | **P Value**^2^ | **Overall**, N = 6,025^1^ | **No**, N = 4,500^1^ | **Yes**, N = 1,525^1^ | **P Value**^2^ |
| **Age (years)** | 57 (51, 63) | 57 (51, 63) | 57 (51, 63) | 0.2 | 57 (51, 63) | 57 (51, 63) | 57 (51, 63) | 0.2 |
| **Gender** |  |  |  | **<0.001** |  |  |  | **<0.001** |
| *Male* | 2,104 (46%) | 1,738 (51%) | 366 (30%) |  | 2,765 (46%) | 2,298 (51%) | 467 (31%) |  |
| *Female* | 2,485 (54%) | 1,650 (49%) | 835 (70%) |  | 3,260 (54%) | 2,202 (49%) | 1,058 (69%) |  |
| **Hukou** |  |  |  | **<0.001** |  |  |  | **<0.001** |
| *Urban* | 615 (13%) | 517 (15%) | 98 (8.2%) |  | 953 (16%) | 795 (18%) | 158 (10%) |  |
| *Rural* | 3,974 (87%) | 2,871 (85%) | 1,103 (92%) |  | 5,072 (84%) | 3,705 (82%) | 1,367 (90%) |  |
| **Household consumption** |  |  |  | 0.2 |  |  |  | **0.017** |
| *< 7700* | 3,447 (75%) | 2,528 (75%) | 919 (77%) |  | 4,423 (73%) | 3,268 (73%) | 1,155 (76%) |  |
| *>= 7700* | 1,142 (25%) | 860 (25%) | 282 (23%) |  | 1,602 (27%) | 1,232 (27%) | 370 (24%) |  |
| **Education** |  |  |  | **<0.001** |  |  |  | **<0.001** |
| *< High school* | 4,150 (90%) | 3,017 (89%) | 1,133 (94%) |  | 5,377 (89%) | 3,953 (88%) | 1,424 (93%) |  |
| *>= High school* | 439 (9.6%) | 371 (11%) | 68 (5.7%) |  | 648 (11%) | 547 (12%) | 101 (6.6%) |  |
| **Marry** |  |  |  | 0.057 |  |  |  | 0.087 |
| *Unmarried* | 412 (9.0%) | 288 (8.5%) | 124 (10%) |  | 558 (9.3%) | 400 (8.9%) | 158 (10%) |  |
| *Married* | 4,177 (91%) | 3,100 (91%) | 1,077 (90%) |  | 5,467 (91%) | 4,100 (91%) | 1,367 (90%) |  |
| **BMI** |  |  |  | 0.2 |  |  |  | 0.14 |
| *Underweight* | 269 (5.9%) | 185 (5.5%) | 84 (7.0%) |  | 344 (5.7%) | 241 (5.4%) | 103 (6.8%) |  |
| *Normal* | 2,412 (53%) | 1,791 (53%) | 621 (52%) |  | 3,151 (52%) | 2,366 (53%) | 785 (51%) |  |
| *Overweight* | 1,374 (30%) | 1,027 (30%) | 347 (29%) |  | 1,815 (30%) | 1,369 (30%) | 446 (29%) |  |
| *Obesity* | 534 (12%) | 385 (11%) | 149 (12%) |  | 715 (12%) | 524 (12%) | 191 (13%) |  |
| **Smoking status** |  |  |  | **<0.001** |  |  |  | **<0.001** |
| *Non_smoker* | 3,187 (69%) | 2,271 (67%) | 916 (76%) |  | 4,226 (70%) | 3,044 (68%) | 1,182 (78%) |  |
| *Smoke* | 1,402 (31%) | 1,117 (33%) | 285 (24%) |  | 1,799 (30%) | 1,456 (32%) | 343 (22%) |  |
| **Drinking status** |  |  |  | **<0.001** |  |  |  | **<0.001** |
| *Non_drinker* | 3,759 (82%) | 2,706 (80%) | 1,053 (88%) |  | 4,965 (82%) | 3,627 (81%) | 1,338 (88%) |  |
| *Drink* | 830 (18%) | 682 (20%) | 148 (12%) |  | 1,060 (18%) | 873 (19%) | 187 (12%) |  |
| **hypertension** |  |  |  | **0.015** |  |  |  | **0.002** |
| *No* | 3,455 (75%) | 2,582 (76%) | 873 (73%) |  | 4,514 (75%) | 3,416 (76%) | 1,098 (72%) |  |
| *Yes* | 1,134 (25%) | 806 (24%) | 328 (27%) |  | 1,511 (25%) | 1,084 (24%) | 427 (28%) |  |
| **Diabetes** |  |  |  | 0.4 |  |  |  | 0.9 |
| *No* | 4,362 (95%) | 3,215 (95%) | 1,147 (96%) |  | 5,714 (95%) | 4,269 (95%) | 1,445 (95%) |  |
| *Yes* | 227 (4.9%) | 173 (5.1%) | 54 (4.5%) |  | 311 (5.2%) | 231 (5.1%) | 80 (5.2%) |  |
| **Heart disease** |  |  |  | **<0.001** |  |  |  | **<0.001** |
| *No* | 4,125 (90%) | 3,122 (92%) | 1,003 (84%) |  | 5,430 (90%) | 4,143 (92%) | 1,287 (84%) |  |
| *Yes* | 464 (10%) | 266 (7.9%) | 198 (16%) |  | 595 (9.9%) | 357 (7.9%) | 238 (16%) |  |
| **Lung disease** |  |  |  | **0.001** |  |  |  | **<0.001** |
| *No* | 4,207 (92%) | 3,133 (92%) | 1,074 (89%) |  | 5,546 (92%) | 4,179 (93%) | 1,367 (90%) |  |
| *Yes* | 382 (8.3%) | 255 (7.5%) | 127 (11%) |  | 479 (8.0%) | 321 (7.1%) | 158 (10%) |  |
| ^1^Median (IQR); n (%) | | | | | | | | |
| ^2^Wilcoxon rank sum test; Pearson's Chi-squared test | | | | | | | | |

**Supplementary Table S2** Body pain status of participants in the second follow-up cycle (wave2-2013) by fuel type.

|  | **Heat** | | | | **Cook** | | | |
| --- | --- | --- | --- | --- | --- | --- | --- | --- |
| **Variable** | **Overall**, N = 11,882^1^ | **clean fuel**, N = 3,606^1^ | **solid fuel**, N = 8,276^1^ | **P Value**^2^ | **Overall**, N = 16,493^1^ | **clean fuel**, N = 8,951^1^ | **solid fuel**, N = 7,542^1^ | **P Value**^2^ |
| **Head_pain** | 1,067 (9.0%) | 233 (6.5%) | 834 (10%) | **<0.001** | 1,359 (8.2%) | 566 (6.3%) | 793 (11%) | **<0.001** |
| **Shoulder_pain** | 1,065 (9.0%) | 233 (6.5%) | 832 (10%) | **<0.001** | 1,395 (8.5%) | 626 (7.0%) | 769 (10%) | **<0.001** |
| **Arm_pain** | 865 (7.3%) | 134 (3.7%) | 731 (8.8%) | **<0.001** | 1,095 (6.6%) | 408 (4.6%) | 687 (9.1%) | **<0.001** |
| **Wrist_pain** | 493 (4.1%) | 87 (2.4%) | 406 (4.9%) | **<0.001** | 617 (3.7%) | 225 (2.5%) | 392 (5.2%) | **<0.001** |
| **Fingers_pain** | 543 (4.6%) | 94 (2.6%) | 449 (5.4%) | **<0.001** | 683 (4.1%) | 258 (2.9%) | 425 (5.6%) | **<0.001** |
| **Chest_pain** | 557 (4.7%) | 106 (2.9%) | 451 (5.4%) | **<0.001** | 714 (4.3%) | 295 (3.3%) | 419 (5.6%) | **<0.001** |
| **Stomach_pain** | 538 (4.5%) | 112 (3.1%) | 426 (5.1%) | **<0.001** | 697 (4.2%) | 297 (3.3%) | 400 (5.3%) | **<0.001** |
| **Back_pain** | 846 (7.1%) | 143 (4.0%) | 703 (8.5%) | **<0.001** | 1,102 (6.7%) | 419 (4.7%) | 683 (9.1%) | **<0.001** |
| **Waist_pain** | 1,924 (16%) | 424 (12%) | 1,500 (18%) | **<0.001** | 2,553 (15%) | 1,121 (13%) | 1,432 (19%) | **<0.001** |
| **Buttocks_pain** | 318 (2.7%) | 53 (1.5%) | 265 (3.2%) | **<0.001** | 399 (2.4%) | 151 (1.7%) | 248 (3.3%) | **<0.001** |
| **Leg_pain** | 1,505 (13%) | 260 (7.2%) | 1,245 (15%) | **<0.001** | 1,978 (12%) | 780 (8.7%) | 1,198 (16%) | **<0.001** |
| **Knees_pain** | 1,161 (9.8%) | 218 (6.0%) | 943 (11%) | **<0.001** | 1,486 (9.0%) | 619 (6.9%) | 867 (11%) | **<0.001** |
| **Ankle_pain** | 575 (4.8%) | 101 (2.8%) | 474 (5.7%) | **<0.001** | 707 (4.3%) | 261 (2.9%) | 446 (5.9%) | **<0.001** |
| **Toes_pain** | 328 (2.8%) | 46 (1.3%) | 282 (3.4%) | **<0.001** | 411 (2.5%) | 146 (1.6%) | 265 (3.5%) | **<0.001** |
| **Neck_pain** | 554 (4.7%) | 97 (2.7%) | 457 (5.5%) | **<0.001** | 722 (4.4%) | 312 (3.5%) | 410 (5.4%) | **<0.001** |
| **multisite pain burden disorders (MPBD)** |  |  |  | **<0.001** |  |  |  | **<0.001** |
| *No* | 11,399 (96%) | 3,541 (98%) | 7,858 (95%) |  | 15,914 (96%) | 8,771 (98%) | 7,143 (95%) |  |
| *Yes* | 483 (4.1%) | 65 (1.8%) | 418 (5.1%) |  | 579 (3.5%) | 180 (2.0%) | 399 (5.3%) |  |
| ^1^n (%) | | | | | | | | |
| ^2^Pearson's Chi-squared test | | | | | | | | |

**Supplementary Table S3** Body pain status of participants in the third follow-up cycle (wave3-2015) by fuel type.

|  | **Heat** | | | | **Cook** | | | |
| --- | --- | --- | --- | --- | --- | --- | --- | --- |
| **Variable** | **Overall**, N = 2,063^1^ | **clean fuel**, N = 613^1^ | **solid fuel**, N = 1,450^1^ | **P Value**^2^ | **Overall**, N = 19,376^1^ | **clean fuel**, N = 11,778^1^ | **solid fuel**, N = 7,598^1^ | **P Value**^2^ |
| **Head_pain** | 196 (9.5%) | 51 (8.3%) | 145 (10%) | 0.2 | 2,806 (14%) | 1,330 (11%) | 1,476 (19%) | **<0.001** |
| **Shoulder_pain** | 221 (11%) | 48 (7.8%) | 173 (12%) | **0.006** | 2,776 (14%) | 1,347 (11%) | 1,429 (19%) | **<0.001** |
| **Arm_pain** | 180 (8.7%) | 44 (7.2%) | 136 (9.4%) | 0.11 | 2,206 (11%) | 1,024 (8.7%) | 1,182 (16%) | **<0.001** |
| **Wrist_pain** | 118 (5.7%) | 27 (4.4%) | 91 (6.3%) | 0.094 | 1,625 (8.4%) | 711 (6.0%) | 914 (12%) | **<0.001** |
| **Fingers_pain** | 135 (6.5%) | 32 (5.2%) | 103 (7.1%) | 0.11 | 1,625 (8.4%) | 716 (6.1%) | 909 (12%) | **<0.001** |
| **Chest_pain** | 95 (4.6%) | 20 (3.3%) | 75 (5.2%) | 0.059 | 1,257 (6.5%) | 567 (4.8%) | 690 (9.1%) | **<0.001** |
| **Stomach_pain** | 127 (6.2%) | 33 (5.4%) | 94 (6.5%) | 0.3 | 1,881 (9.7%) | 896 (7.6%) | 985 (13%) | **<0.001** |
| **Back_pain** | 178 (8.6%) | 35 (5.7%) | 143 (9.9%) | **0.002** | 2,154 (11%) | 990 (8.4%) | 1,164 (15%) | **<0.001** |
| **Waist_pain** | 305 (15%) | 78 (13%) | 227 (16%) | 0.087 | 3,848 (20%) | 1,895 (16%) | 1,953 (26%) | **<0.001** |
| **Buttocks_pain** | 69 (3.3%) | 14 (2.3%) | 55 (3.8%) | 0.081 | 1,104 (5.7%) | 503 (4.3%) | 601 (7.9%) | **<0.001** |
| **Leg_pain** | 237 (11%) | 65 (11%) | 172 (12%) | 0.4 | 2,845 (15%) | 1,292 (11%) | 1,553 (20%) | **<0.001** |
| **Knees_pain** | 248 (12%) | 58 (9.5%) | 190 (13%) | **0.020** | 2,970 (15%) | 1,379 (12%) | 1,591 (21%) | **<0.001** |
| **Ankle_pain** | 116 (5.6%) | 22 (3.6%) | 94 (6.5%) | **0.009** | 1,497 (7.7%) | 656 (5.6%) | 841 (11%) | **<0.001** |
| **Toes_pain** | 68 (3.3%) | 16 (2.6%) | 52 (3.6%) | 0.3 | 980 (5.1%) | 409 (3.5%) | 571 (7.5%) | **<0.001** |
| **Neck_pain** | 178 (8.6%) | 35 (5.7%) | 143 (9.9%) | **0.002** | 1,844 (9.5%) | 908 (7.7%) | 936 (12%) | **<0.001** |
| **multisite pain burden disorders (MPBD)** |  |  |  | **0.006** |  |  |  | **<0.001** |
| *No* | 1,896 (92%) | 579 (94%) | 1,317 (91%) |  | 17,346 (90%) | 10,876 (92%) | 6,470 (85%) |  |
| *Yes* | 167 (8.1%) | 34 (5.5%) | 133 (9.2%) |  | 2,030 (10%) | 902 (7.7%) | 1,128 (15%) |  |
| ^1^n (%) | | | | | | | | |
| ^2^Pearson's Chi-squared test | | | | | | | | |

**Supplementary Table S4** Body pain status of participants in the fourth follow-up cycle (wave4-2018) by fuel type.

|  | **Heat** | | | | **Cook** | | | |
| --- | --- | --- | --- | --- | --- | --- | --- | --- |
| **Variable** | **Overall**, N = 1,541^1^ | **clean fuel**, N = 560^1^ | **solid fuel**, N = 981^1^ | **P Value**^2^ | **Overall**, N = 19,262^1^ | **clean fuel**, N = 13,296^1^ | **solid fuel**, N = 5,966^1^ | **P Value**^2^ |
| **Head_pain** | 381 (25%) | 108 (19%) | 273 (28%) | **<0.001** | 4,883 (25%) | 2,941 (22%) | 1,942 (33%) | **<0.001** |
| **Shoulder_pain** | 387 (25%) | 117 (21%) | 270 (28%) | **0.004** | 5,092 (26%) | 3,216 (24%) | 1,876 (31%) | **<0.001** |
| **Arm_pain** | 278 (18%) | 90 (16%) | 188 (19%) | 0.13 | 3,862 (20%) | 2,298 (17%) | 1,564 (26%) | **<0.001** |
| **Wrist_pain** | 204 (13%) | 51 (9.1%) | 153 (16%) | **<0.001** | 2,621 (14%) | 1,512 (11%) | 1,109 (19%) | **<0.001** |
| **Fingers_pain** | 214 (14%) | 58 (10%) | 156 (16%) | **0.002** | 2,788 (14%) | 1,618 (12%) | 1,170 (20%) | **<0.001** |
| **Chest_pain** | 144 (9.3%) | 40 (7.1%) | 104 (11%) | **0.025** | 2,036 (11%) | 1,150 (8.6%) | 886 (15%) | **<0.001** |
| **Stomach_pain** | 237 (15%) | 59 (11%) | 178 (18%) | **<0.001** | 3,234 (17%) | 1,963 (15%) | 1,271 (21%) | **<0.001** |
| **Back_pain** | 315 (20%) | 89 (16%) | 226 (23%) | **<0.001** | 3,681 (19%) | 2,165 (16%) | 1,516 (25%) | **<0.001** |
| **Waist_pain** | 535 (35%) | 168 (30%) | 367 (37%) | **0.003** | 7,177 (37%) | 4,543 (34%) | 2,634 (44%) | **<0.001** |
| **Buttocks_pain** | 121 (7.9%) | 34 (6.1%) | 87 (8.9%) | **0.050** | 1,785 (9.3%) | 1,021 (7.7%) | 764 (13%) | **<0.001** |
| **Leg_pain** | 357 (23%) | 104 (19%) | 253 (26%) | **0.001** | 4,917 (26%) | 2,897 (22%) | 2,020 (34%) | **<0.001** |
| **Knees_pain** | 426 (28%) | 131 (23%) | 295 (30%) | **0.005** | 5,533 (29%) | 3,380 (25%) | 2,153 (36%) | **<0.001** |
| **Ankle_pain** | 199 (13%) | 54 (9.6%) | 145 (15%) | **0.004** | 2,583 (13%) | 1,443 (11%) | 1,140 (19%) | **<0.001** |
| **Toes_pain** | 124 (8.0%) | 29 (5.2%) | 95 (9.7%) | **0.002** | 1,769 (9.2%) | 968 (7.3%) | 801 (13%) | **<0.001** |
| **Neck_pain** | 302 (20%) | 86 (15%) | 216 (22%) | **0.002** | 3,502 (18%) | 2,220 (17%) | 1,282 (21%) | **<0.001** |
| **multisite pain burden disorders (MPBD)** |  |  |  | **<0.001** |  |  |  | **<0.001** |
| *No* | 1,279 (83%) | 489 (87%) | 790 (81%) |  | 15,974 (83%) | 11,429 (86%) | 4,545 (76%) |  |
| *Yes* | 262 (17%) | 71 (13%) | 191 (19%) |  | 3,288 (17%) | 1,867 (14%) | 1,421 (24%) |  |
| ^1^n (%) | | | | | | | | |
| ^2^Pearson's Chi-squared test | | | | | | | | |

**Supplementary Table S5** Body pain status of participants in the fifth follow-up cycle (wave5-2020) by fuel type.

|  | **Heat** | | | | **Cook** | | | |
| --- | --- | --- | --- | --- | --- | --- | --- | --- |
| **Variable** | **Overall**, N = 12,336^1^ | **clean fuel**, N = 6,060^1^ | **solid fuel**, N = 6,276^1^ | **P Value**^2^ | **Overall**, N = 18,970^1^ | **clean fuel**, N = 13,995^1^ | **solid fuel**, N = 4,975^1^ | **P Value**^2^ |
| **Head_pain** | 2,711 (22%) | 1,083 (18%) | 1,628 (26%) | **<0.001** | 3,941 (21%) | 2,581 (18%) | 1,360 (27%) | **<0.001** |
| **Shoulder_pain** | 2,698 (22%) | 1,206 (20%) | 1,492 (24%) | **<0.001** | 3,924 (21%) | 2,671 (19%) | 1,253 (25%) | **<0.001** |
| **Arm_pain** | 2,104 (17%) | 846 (14%) | 1,258 (20%) | **<0.001** | 3,004 (16%) | 1,930 (14%) | 1,074 (22%) | **<0.001** |
| **Wrist_pain** | 1,366 (11%) | 550 (9.1%) | 816 (13%) | **<0.001** | 1,959 (10%) | 1,237 (8.8%) | 722 (15%) | **<0.001** |
| **Fingers_pain** | 1,580 (13%) | 610 (10%) | 970 (15%) | **<0.001** | 2,262 (12%) | 1,421 (10%) | 841 (17%) | **<0.001** |
| **Chest_pain** | 1,130 (9.2%) | 425 (7.0%) | 705 (11%) | **<0.001** | 1,635 (8.6%) | 1,029 (7.4%) | 606 (12%) | **<0.001** |
| **Stomach_pain** | 1,886 (15%) | 774 (13%) | 1,112 (18%) | **<0.001** | 2,673 (14%) | 1,725 (12%) | 948 (19%) | **<0.001** |
| **Back_pain** | 1,841 (15%) | 694 (11%) | 1,147 (18%) | **<0.001** | 2,667 (14%) | 1,706 (12%) | 961 (19%) | **<0.001** |
| **Waist_pain** | 4,167 (34%) | 1,830 (30%) | 2,337 (37%) | **<0.001** | 6,159 (32%) | 4,255 (30%) | 1,904 (38%) | **<0.001** |
| **Buttocks_pain** | 1,078 (8.7%) | 436 (7.2%) | 642 (10%) | **<0.001** | 1,532 (8.1%) | 961 (6.9%) | 571 (11%) | **<0.001** |
| **Leg_pain** | 3,100 (25%) | 1,190 (20%) | 1,910 (30%) | **<0.001** | 4,452 (23%) | 2,901 (21%) | 1,551 (31%) | **<0.001** |
| **Knees_pain** | 3,288 (27%) | 1,365 (23%) | 1,923 (31%) | **<0.001** | 4,832 (25%) | 3,192 (23%) | 1,640 (33%) | **<0.001** |
| **Ankle_pain** | 1,441 (12%) | 555 (9.2%) | 886 (14%) | **<0.001** | 2,101 (11%) | 1,310 (9.4%) | 791 (16%) | **<0.001** |
| **Toes_pain** | 932 (7.6%) | 343 (5.7%) | 589 (9.4%) | **<0.001** | 1,369 (7.2%) | 836 (6.0%) | 533 (11%) | **<0.001** |
| **Neck_pain** | 1,915 (16%) | 807 (13%) | 1,108 (18%) | **<0.001** | 2,788 (15%) | 1,889 (13%) | 899 (18%) | **<0.001** |
| **multisite pain burden disorders (MPBD)** |  |  |  | **<0.001** |  |  |  | **<0.001** |
| *No* | 10,676 (87%) | 5,439 (90%) | 5,237 (83%) |  | 16,608 (88%) | 12,548 (90%) | 4,060 (82%) |  |
| *Yes* | 1,660 (13%) | 621 (10%) | 1,039 (17%) |  | 2,362 (12%) | 1,447 (10%) | 915 (18%) |  |
| ^1^n (%) | | | | | | | | |
| ^2^Pearson's Chi-squared test | | | | | | | | |

**Supplementary Table S6** Univariate COX analysis between clinical characteristics and the risk of MPBD

|  | **Heat** | | | **Cook** | | |
| --- | --- | --- | --- | --- | --- | --- |
| **Characteristic** | **HR**^1^ | **95% CI**^1^ | **p-value** | **HR**^1^ | **95% CI**^1^ | **p-value** |
| **age** | 1.01 | 1.00, 1.01 | 0.12 | 1.00 | 1.00, 1.01 | 0.2 |
| **gender** |  |  |  |  |  |  |
| *Male* | — | — |  | — | — |  |
| *Female* | 2.13 | 1.88, 2.41 | **<0.001** | 2.11 | 1.89, 2.35 | **<0.001** |
| **hukou** |  |  |  |  |  |  |
| *Urban* | — | — |  | — | — |  |
| *Rural* | 1.89 | 1.53, 2.32 | **<0.001** | 1.74 | 1.48, 2.05 | **<0.001** |
| **household**  **consumption** |  |  |  |  |  |  |
| *< 7700* | — | — |  | — | — |  |
| *>= 7700* | 0.91 | 0.80, 1.04 | 0.2 | 0.86 | 0.77, 0.97 | **0.015** |
| **education** |  |  |  |  |  |  |
| *< High school* | — | — |  | — | — |  |
| *>= High school* | 0.53 | 0.41, 0.67 | **<0.001** | 0.55 | 0.45, 0.67 | **<0.001** |
| **marry** |  |  |  |  |  |  |
| *Unmarried* | — | — |  | — | — |  |
| *Married* | 0.83 | 0.69, 1.00 | 0.056 | 0.86 | 0.73, 1.02 | 0.081 |
| **bmi** |  |  |  |  |  |  |
| *Underweight* | — | — |  | — | — |  |
| *Normal* | 0.78 | 0.62, 0.98 | **0.032** | 0.80 | 0.65, 0.98 | **0.030** |
| *Overweight* | 0.76 | 0.60, 0.96 | **0.022** | 0.78 | 0.63, 0.97 | **0.023** |
| *Obesity* | 0.86 | 0.66, 1.12 | 0.3 | 0.87 | 0.68, 1.10 | 0.2 |
| **smoke** |  |  |  |  |  |  |
| *Non_smoker* | — | — |  | — | — |  |
| *Smoke* | 0.67 | 0.59, 0.77 | **<0.001** | 0.65 | 0.57, 0.73 | **<0.001** |
| **drink** |  |  |  |  |  |  |
| *Non_drinker* | — | — |  | — | — |  |
| *Drink* | 0.60 | 0.51, 0.71 | **<0.001** | 0.62 | 0.53, 0.72 | **<0.001** |
| **hypertension** |  |  |  |  |  |  |
| *No* | — | — |  | — | — |  |
| *Yes* | 1.19 | 1.05, 1.35 | **0.008** | 1.21 | 1.08, 1.35 | **<0.001** |
| **diabetes** |  |  |  |  |  |  |
| *No* | — | — |  | — | — |  |
| *Yes* | 0.88 | 0.67, 1.16 | 0.4 | 1.02 | 0.81, 1.28 | 0.9 |
| **heart_disease** |  |  |  |  |  |  |
| *No* | — | — |  | — | — |  |
| *Yes* | 2.02 | 1.74, 2.36 | **<0.001** | 1.91 | 1.67, 2.20 | **<0.001** |
| **lung_disease** |  |  |  |  |  |  |
| *No* | — | — |  | — | — |  |
| *Yes* | 1.39 | 1.16, 1.67 | **<0.001** | 1.43 | 1.22, 1.69 | **<0.001** |
| ^1^HR = Hazard Ratio, CI = Confidence Interval | | | | | | |

**Supplementary Table S7** Hazard ratios of MPBD associated with the use of solid fuels for heating or cooking (threshold ≥4).

|  | model1 | | | model2 | | | model3 | | |
| --- | --- | --- | --- | --- | --- | --- | --- | --- | --- |
| **Characteristic** | **HR**^1^ | **95% CI**^1^ | **p-value** | **HR**^1^ | **95% CI**^1^ | **p-value** | **HR**^1^ | **95% CI**^1^ | **p-value** |
| **heat_fuel** |  |  |  |  |  |  |  |  |  |
| *clean fuel heat* | — | — |  | — | — |  | — | — |  |
| *solid fuel heat* | 1.27 | 1.13, 1.42 | **<0.001** | 1.21 | 1.07, 1.36 | **0.002** | 1.20 | 1.06, 1.35 | **0.003** |
| **cook_fuel** |  |  |  |  |  |  |  |  |  |
| *clean fuel cook* | — | — |  | — | — |  | — | — |  |
| *solid fuel cook* | 1.20 | 1.11, 1.31 | **<0.001** | 1.12 | 1.03, 1.23 | **0.011** | 1.12 | 1.03, 1.23 | **0.011** |
| ^1^HR = Hazard Ratio, CI = Confidence Interval | | | | | | | | | |

Model 1: Unadjusted. Model 2: Adjusted for age, gender, hukou, household consumption, education, and marital status. Model 3: Adjusted for age, gender, hukou, household consumption, education, marital status, BMI, smoking, drinking, hypertension, heart disease, diabetes, and lung diseases. P < 0.05 is highlighted in bold.

**Supplementary Table S8** Hazard ratios of MPBD associated with the use of solid fuels for heating or cooking (threshold ≥10).

|  | model1 | | | model2 | | | model3 | | |
| --- | --- | --- | --- | --- | --- | --- | --- | --- | --- |
| **Characteristic** | **HR**^1^ | **95% CI**^1^ | **p-value** | **HR**^1^ | **95% CI**^1^ | **p-value** | **HR**^1^ | **95% CI**^1^ | **p-value** |
| **heat_fuel** |  |  |  |  |  |  |  |  |  |
| *clean fuel heat* | — | — |  | — | — |  | — | — |  |
| *solid fuel heat* | 1.66 | 1.35, 2.05 | **<0.001** | 1.50 | 1.21, 1.86 | **<0.001** | 1.46 | 1.18, 1.80 | **<0.001** |
| **cook_fuel** |  |  |  |  |  |  |  |  |  |
| *clean fuel cook* | — | — |  | — | — |  | — | — |  |
| *solid fuel cook* | 1.53 | 1.33, 1.77 | **<0.001** | 1.37 | 1.17, 1.59 | **<0.001** | 1.35 | 1.16, 1.57 | **<0.001** |
| ^1^HR = Hazard Ratio, CI = Confidence Interval | | | | | | | | | |

Model 1: Unadjusted. Model 2: Adjusted for age, gender, hukou, household consumption, education, and marital status. Model 3: Adjusted for age, gender, hukou, household consumption, education, marital status, BMI, smoking, drinking, hypertension, heart disease, diabetes, and lung diseases. P < 0.05 is highlighted in bold.

**Supplementary Table S9** Distribution characteristics of baseline variables between the excluded and included groups.

|  | **Heating Fuel** | | | **Cooking Fuel** | | |
| --- | --- | --- | --- | --- | --- | --- |
| **Variable** | **Excluded**, N = 1,983^1^ | **Included**, N = 4,589^1^ | **P Value**^2^ | **Excluded**, N = 2,764^1^ | **Included**, N = 6,025^1^ | **P Value**^2^ |
| **Age (years)** | 57.3 (8.2) | 57.5 (8.2) | 0.4 | 57.6 (8.4) | 57.6 (8.3) | 0.8 |
| **Gender** |  |  | **<0.001** |  |  | **<0.001** |
| *Male* | 1,049 (53%) | 2,104 (46%) |  | 1,451 (52%) | 2,765 (46%) |  |
| *Female* | 934 (47%) | 2,485 (54%) |  | 1,313 (48%) | 3,260 (54%) |  |
| **Residence** |  |  | **<0.001** |  |  | **<0.001** |
| *Urban* | 339 (17%) | 615 (13%) |  | 586 (21%) | 953 (16%) |  |
| *Rural* | 1,643 (83%) | 3,974 (87%) |  | 2,175 (79%) | 5,072 (84%) |  |
| **Household consumption** |  |  | **0.001** |  |  | **<0.001** |
| *< 7700* | 786 (70%) | 3,447 (75%) |  | 987 (67%) | 4,423 (73%) |  |
| *>= 7700* | 330 (30%) | 1,142 (25%) |  | 490 (33%) | 1,602 (27%) |  |
| **Education level** |  |  | **0.001** |  |  | **<0.001** |
| *< High school* | 1,740 (88%) | 4,150 (90%) |  | 2,398 (87%) | 5,377 (89%) |  |
| *>= High school* | 243 (12%) | 439 (9.6%) |  | 366 (13%) | 648 (11%) |  |
| **Marital status** |  |  | 0.2 |  |  | 0.3 |
| *Unmarried* | 200 (10%) | 412 (9.0%) |  | 275 (9.9%) | 558 (9.3%) |  |
| *Married* | 1,783 (90%) | 4,177 (91%) |  | 2,489 (90%) | 5,467 (91%) |  |
| **BMI category** |  |  | >0.9 |  |  | >0.9 |
| *Underweight* | 1,008 (100%) | 4,589 (100%) |  | 1,437 (100%) | 6,025 (100%) |  |
| *Normal* | 0 (0%) | 0 (0%) |  | 0 (0%) | 0 (0%) |  |
| *Overweight* | 0 (0%) | 0 (0%) |  | 0 (0%) | 0 (0%) |  |
| *Obesity* | 0 (0%) | 0 (0%) |  | 0 (0%) | 0 (0%) |  |
| **Smoking** |  |  | 0.5 |  |  | 0.4 |
| *No* | 1,256 (69%) | 3,187 (69%) |  | 1,787 (69%) | 4,226 (70%) |  |
| *Yes* | 576 (31%) | 1,402 (31%) |  | 791 (31%) | 1,799 (30%) |  |
| **Drinking** |  |  | **0.019** |  |  | 0.050 |
| *No* | 1,326 (85%) | 3,759 (82%) |  | 1,860 (84%) | 4,965 (82%) |  |
| *Yes* | 243 (15%) | 830 (18%) |  | 348 (16%) | 1,060 (18%) |  |
| **Hypertension** |  |  | 0.8 |  |  | 0.7 |
| *No* | 1,470 (76%) | 3,455 (75%) |  | 2,046 (75%) | 4,514 (75%) |  |
| *Yes* | 474 (24%) | 1,134 (25%) |  | 669 (25%) | 1,511 (25%) |  |
| **Diabetes** |  |  | 0.8 |  |  | 0.3 |
| *No* | 1,816 (95%) | 4,362 (95%) |  | 2,530 (94%) | 5,714 (95%) |  |
| *Yes* | 97 (5.1%) | 227 (4.9%) |  | 152 (5.7%) | 311 (5.2%) |  |
| **Heart disease** |  |  | 0.6 |  |  | 0.6 |
| *No* | 1,751 (90%) | 4,125 (90%) |  | 2,453 (91%) | 5,430 (90%) |  |
| *Yes* | 187 (9.6%) | 464 (10%) |  | 257 (9.5%) | 595 (9.9%) |  |
| **Lung disease** |  |  | 0.8 |  |  | 0.7 |
| *No* | 1,793 (92%) | 4,207 (92%) |  | 2,519 (92%) | 5,546 (92%) |  |
| *Yes* | 158 (8.1%) | 382 (8.3%) |  | 210 (7.7%) | 479 (8.0%) |  |
| **Pain burden** |  |  | 0.3 |  |  | 0.5 |
| *No* | 1,487 (75%) | 3,388 (74%) |  | 2,084 (75%) | 4,500 (75%) |  |
| *Yes* | 496 (25%) | 1,201 (26%) |  | 680 (25%) | 1,525 (25%) |  |
| ^1^Mean (SD); n (%) | | | | | | |
| ^2^Wilcoxon rank sum test; Pearson's Chi-squared test; Fisher's exact test | | | | | | |

**Supplementary Table S10** Sensitivity analyses including complete-case analysis, multiple imputation, and inverse probability of censoring weighting (IPCW).

| **Method** | **Fuel_Type** | **Sample_Size** | **HR (95% CI)** | **Relative_Diff_Formatted** |
| --- | --- | --- | --- | --- |
| Complete Case | Heating | 4,589 | 1.21 (1.05-1.41) | - |
| Multiple Imputation | Heating | 6,572 | 1.29 (1.14-1.46) | 6.0% |
| IPCW | Heating | 4,589 | 1.21 (1.04-1.40) | -0.5% |
| Complete Case | Cooking | 6,025 | 1.20 (1.08-1.34) | - |
| Multiple Imputation | Cooking | 8,789 | 1.32 (1.21-1.45) | 10.2% |
| IPCW | Cooking | 6,025 | 1.20 (1.08-1.35) | 0.2% |

**Supplementary Table S11** Sensitivity analysis using the midpoint imputation method between waves.

| **Fuel Type** | **Time Assignment Method** | **Sample Size** | **HR (95% CI)** | **Relative Difference** |
| --- | --- | --- | --- | --- |
| Heating | Original Interview Time | 4,589 | 1.21 (1.05-1.41) | - |
| Heating | Midpoint between Waves | 4,589 | 1.21 (1.05-1.41) | 0.0% |
| Cooking | Original Interview Time | 6,025 | 1.20 (1.08-1.34) | - |
| Cooking | Midpoint between Waves | 6,025 | 1.20 (1.08-1.34) | 0.0% |

**Supplementary Table S12** Proportional hazards assumption tests for solid fuel use in heating and cooking.

| **Heat** | | | | **Cook** | | | |
| --- | --- | --- | --- | --- | --- | --- | --- |
| **Variable** | **Chi-square** | **DF** | **P-value** | **Variable** | **Chi-square** | **DF** | **P-value** |
| Heat fuel | 5.82304 | 1 | 0.0158 | Cook fuel | 15.3053 | 1 | <0.001 |
| Age | 6.89605 | 1 | 0.0086 | Age | 7.0503 | 1 | 0.0079 |
| Gender | 1.87695 | 1 | 0.1707 | Gender | 1.6147 | 1 | 0.2038 |
| Hukou | 1.13414 | 1 | 0.2869 | Hukou | 0.6461 | 1 | 0.4215 |
| Household consumption | 0.42514 | 1 | 0.5144 | Household consumption | 0.4968 | 1 | 0.4809 |
| Education level | 0.00306 | 1 | 0.9559 | Education level | 0.2993 | 1 | 0.5843 |
| Marital status | 0.01684 | 1 | 0.8967 | Marital status | 0.0307 | 1 | 0.8610 |
| BMI | 0.87696 | 1 | 0.349 | BMI | 0.2966 | 1 | 0.5860 |
| Smoking | 0.34963 | 1 | 0.5543 | Smoking | 1.0049 | 1 | 0.3161 |
| Drinking | 0.88258 | 1 | 0.3475 | Drinking | 0.1685 | 1 | 0.6814 |
| Hypertension | 3.32351 | 1 | 0.0683 | Hypertension | 5.2637 | 1 | 0.0218 |
| Diabetes | 1.53982 | 1 | 0.2146 | Diabetes | 0.0549 | 1 | 0.8147 |
| Heart disease | 1.1671 | 1 | 0.28 | Heart disease | 1.6218 | 1 | 0.2028 |
| Lung disease | 1.75545 | 1 | 0.1852 | Lung disease | 1.5541 | 1 | 0.2125 |
| GLOBAL test | 22.15162 | 14 | 0.0756 | GLOBAL test | 27.4973 | 14 | **0.0166** |

**Supplementary Table S13** Time-interaction analysis of the interaction between solid fuel use and the logarithm of follow-up time.

| **Fuel Type** | **Interaction Term** | **Coefficient** | **Hazard Ratio (HR)** | **Standard Error** | **Z-value** | **P-value** |
| --- | --- | --- | --- | --- | --- | --- |
| Heating | Solid fuel × log(time) | -92.73 | 5.35e-41 | 2631.98 | -0.035 | 0.972 |
| Cooking | Solid fuel × log(time) | -93.98 | 1.54e-41 | 2616.21 | -0.036 | 0.971 |

**Supplementary Table S14** Sensitivity analyses incorporating the standard Cox model, survey-weighted models, and cluster-robust standard errors.

| **Variance Estimation Method** | **Heating Fuel HR (95% CI)** | **Cooking Fuel HR (95% CI)** |
| --- | --- | --- |
| Standard Cox | 1.21 (1.05-1.41) | 1.20 (1.08-1.34) |
| Survey-weighted Cox | 1.23 (1.01-1.49) | 1.17 (0.99-1.38) |
| Robust SE (clustered) | 1.21 (1.02-1.45) | 1.20 (1.04-1.38) |

**Supplementary Table S15** Calculation of E-values to assess unmeasured confounding.

| **Analysis** | **Assumption** | **HR** | **CI_95** | **E_value_point** | **E_value_CI** | **Outcome_rate** |
| --- | --- | --- | --- | --- | --- | --- |
| Heating | Rare outcome | 1.212 | (1.017-1.444) | 1.21 | 1.72 | 0.262 |
| Heating | Common outcome | 1.212 | (1.017-1.444) | 1.14 | 1.55 | 0.262 |
| Cooking | Rare outcome | 1.201 | (1.044-1.382) | 1.2 | 1.69 | 0.253 |
| Cooking | Common outcome | 1.201 | (1.044-1.382) | 1.14 | 1.53 | 0.253 |


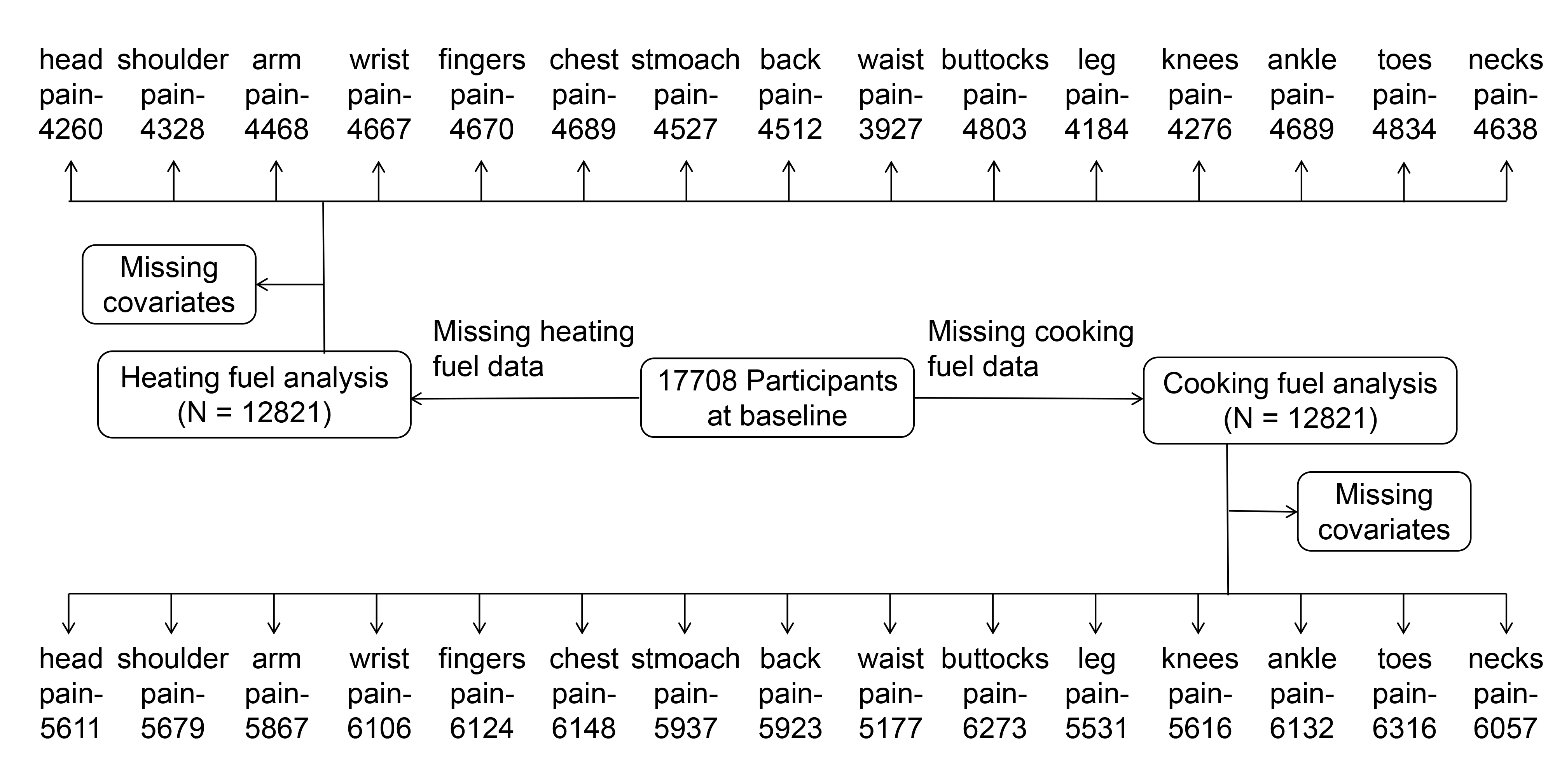


**Supplementary Figure S1** Flowchart of participant inclusion for the analysis of 15 different body pain locations.
